# Peripapillary retinal splitting visualized on OCT in glaucoma and glaucoma suspect patients

**DOI:** 10.1371/journal.pone.0182816

**Published:** 2017-08-23

**Authors:** Dilraj S. Grewal, Daniel J. Merlau, Pushpanjali Giri, Marion R. Munk, Amani A. Fawzi, Lee M. Jampol, Angelo P. Tanna

**Affiliations:** Department of Ophthalmology, Northwestern University Feinberg School of Medicine, Chicago, Illinois, United States of America; Massachusetts Eye & Ear Infirmary, Harvard Medical School, UNITED STATES

## Abstract

**Purpose:**

To identify the risk factors for development of peripapillary retinal splitting (schisis) in patients with glaucoma or suspicion of glaucoma

**Setting:**

Glaucoma Clinic, Department of Ophthalmology, Northwestern University Feinberg School of Medicine, Chicago, IL

**Methods:**

In this institutional cross-sectional study, 495 patients (990 eyes) who had undergone spectral-domain optical coherence tomography (OCT Spectralis HRA-OCT, Heidelberg Engineering) optic nerve head (ONH) imaging and did not have identifiable optic nerve pits, pseudopits or coloboma were included. OCT scans were reviewed by two observers.

**Main outcome measures:**

Presence of peripapillary retinal splitting identified on OCT raster scans.

**Results:**

Eleven of 990 glaucoma and glaucoma suspect eyes (1.1%) of 7 patients (2 females, 5 males, mean age 64.5 ± 9.2 years) had peripapillary retinal splitting. Two of these 11 eyes had extension of the splitting into the macula but none to the fovea. Of these 11 patients, 2 (28.6%) were glaucoma suspects, 3 (42.9%) had primary open-angle glaucoma, 1 (14.3%) had chronic angle-closure glaucoma and 1 (14.3%) had pigmentary glaucoma. 7/11 (63.6%) eyes had vitreous traction to the disc visualized on OCT and 6/11 eyes (54.5%) had beta-zone peripapillary atrophy.

**Conclusions:**

We observed peripapillary retinal splitting in 1.1% of a series of 990 glaucoma and glaucoma-suspect eyes. Evidence of adherent vitreous with traction and peripapillary atrophy was found in a majority of the involved eyes. A comparison to an age and axial length matched cohort is required to determine if this is a condition that is associated with glaucoma.

## Introduction

Peripapillary retinal splitting (retinoschisis), which is characterized by the splitting of the peripapillary retinal nerve fiber layer (RNFL), inner or outer plexiform and/or nuclear retinal layers with schisis cavities, occurs in eyes with congenital cavitary optic disc anomalies (CODA) including, colobomas or pits and morning glory syndrome. [1–4] It has been previously reported in patients with glaucoma without optic disc pits. [5–8] There has also been increased interest in peripapillary and macular retinoschisis in glaucomatous eyes recently. [9–11] The impact of retinoschisis on RNFL thickness measurements has been noted illustrating its effect as a confounding factor in detecting structural progression in glacuoma. [11]

There are no previous reports of the prevalance of peripapillary retinoschsiis in glaucoma and glaucoma suspects. In addition, most of the previous reports were based on isolated cases or case series with no identification of factors associated with the presence of peripapillary splitting. In the current study, we used high density optical coherence tomography (OCT) raster scans of the optic nerve head to identify the prevalence of peripapillary splitting, peripapillary atrophy and vitreous traction in a large series of patients with glaucoma or suspicion of glaucoma.

## Methods

The study was approved by the Institutional Review Board at Northwestern University, Feinberg School of Medicine and adhered to the tenets set forth by the Declaration of Helsinki. The Institutional Review Board waived the requirement for written informed consent.

We analyzed the medical records of consecutive patients examined in our glaucoma clinic over a period of 3 months at the Department of Ophthalmology, Northwestern University Feinberg School of Medicine. Exclusion criteria were the presence of CODA, optic nerve pits, pseudopits or coloboma, concurrent retinal disease (for example, vascular disorders like central retinal vein occlusion (CRVO), branch retinal vein occlusion (BRVO), or macular degeneration), optic nerve disease other than glaucoma or media opacity precluding a good quality OCT scan.

Optic nerve head scans of subjects with glaucoma or suspicion of glaucoma underwent spectral domain OCT imaging. The raster lines protocol (4 mm by 4 mm area centered on the optic disc of the Spectralis HRA-OCT was used (Version 4; Heidelberg Engineering, Heidelberg, Germany). The standard raster volume scan (61 B-scans with 125 um inter-scan spacing) was performed. OCT image quality was identified from the quality bar (range 0–40 decibels). Scans with artifacts or scan quality <15 dB were excluded. [12]

OCT data were exported from the imaging instruments and B-scans for each slice were reviewed. Scans with poor quality were excluded. OCT scans for both groups were reviewed by two independent observers. The two observers independently reviewed the individual B scans for both groups to detect the presence of schisis. The main outcome measure was the presence of peripapillary schisis identified on OCT raster scans. The splitting included any eyes with splitting delineated by cystoid spaces in the retinal nerve fiber layer (RNFL), inner nuclear layer (INL), outer nuclear layer (ONL) and outer plexiform layer (OPL). All cases of schisis were verified by a team of a two retina specialists (AAF and LMJ) and one glaucoma specialist (APT). Cases with uncertain findings on the initial evaluation were adjudicated by the same team.

As part of their routine follow-up, each subject underwent a full ophthalmic examination, including assessment of visual acuity, intraocular pressure (IOP) with a Goldmann applanation tonometer, optic nerve head (ONH) evaluation and stereoscopic slit lamp biomicroscopic fundus examination with a 78 or 90-diopter lens, and the 24–2 Swedish Interactive Threshold Algorithm standard automated visual field (VF) test (Humphrey VF Analyzer; Carl Zeiss Meditec, Dublin, California, USA).

## Statistical analysis

Statistical analysis was performed with Statistical Package for the Social Sciences V.20.0 (SPSS, Chicago, Illinois, USA). Data were analyzed descriptively and are given in mean ± standard deviation (SD) and in frequencies. Chi square test was used for comparisons between cases of peripapillary splitting among glaucoma and glaucoma suspects and controls. Independent samples t-test was used for continuous variables. *P* values <0.05 were considered statistically significant.

## Results

**Fig 1:**
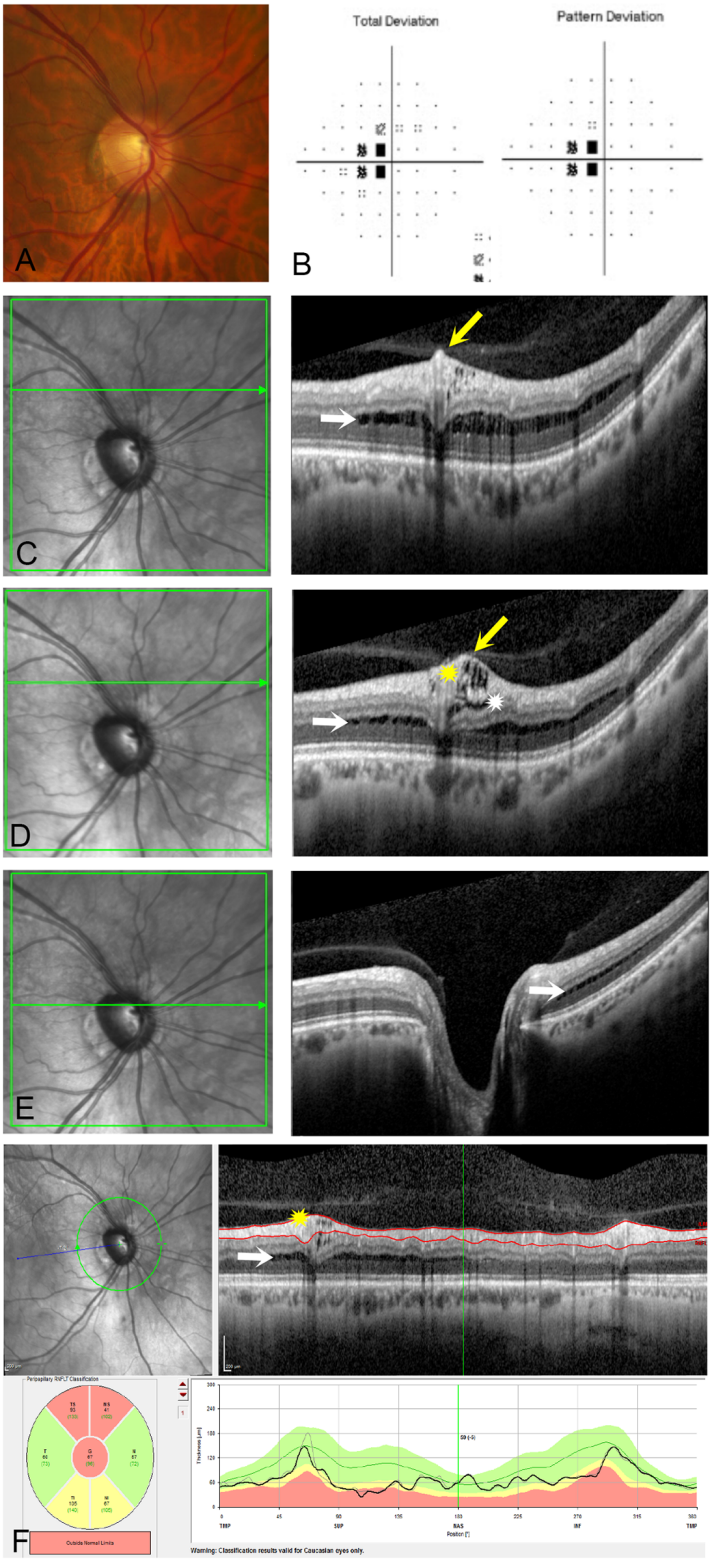
78 year old male with primary open angle glaucoma (POAG) with peripapillary retinoschisis. (1A) Optic nerve photograph shows glaucomatous optic neuropathy (1B) There is mild visual field damage with a mean deviation of -2.19 dB. (1C, 1D and 1E) Three horizontal OCT raster scans through three different sections of the optic nerve demonstrating peripapillary retinal splitting (retinoschisis) with adherent vitreous in the region of retinoschisis (yellow arrow), splitting in the nerve fiber layer (yellow star), and inner plexiform layer (white star) and outer plexiform layer with a likely outer nuclear layer component as well (white arrows). (1F) Circumpapillary retinal nerve fiber layer (RNFL) thickness map shows retinoschisis nasally within the RNFL segmentation (yellow star), causing a small area of artifactual thickening on the RNFL thickness profile.

**Fig 2:**
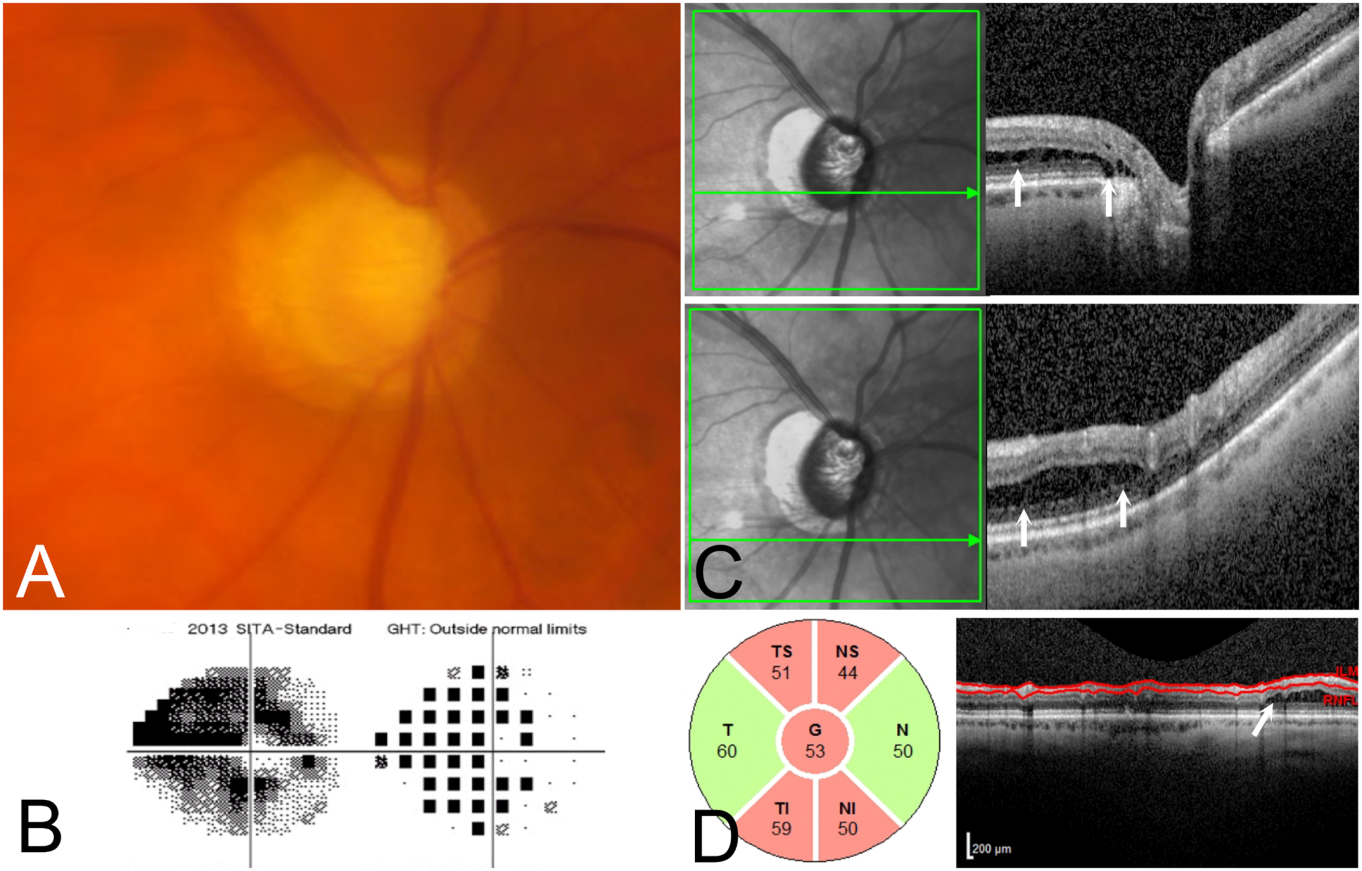
67 year old male with advanced POAG, with peripapillary retinochisis. (2A) Optic nerve photograph shows glaucomatous optic neuropathy. (2B) There is advanced visual field damage, with a mean deviation of -19.65 dB. (2C) OCT demonstrates peripapillary retinoschisis in the outer nuclear layer and outer plexiform layer (C; white arrows) on two horizontal raster scans through the optic nerve. (2D) Circumpapillary RNFL thickness map shows retinoschisis nasally.

**Fig 3:**
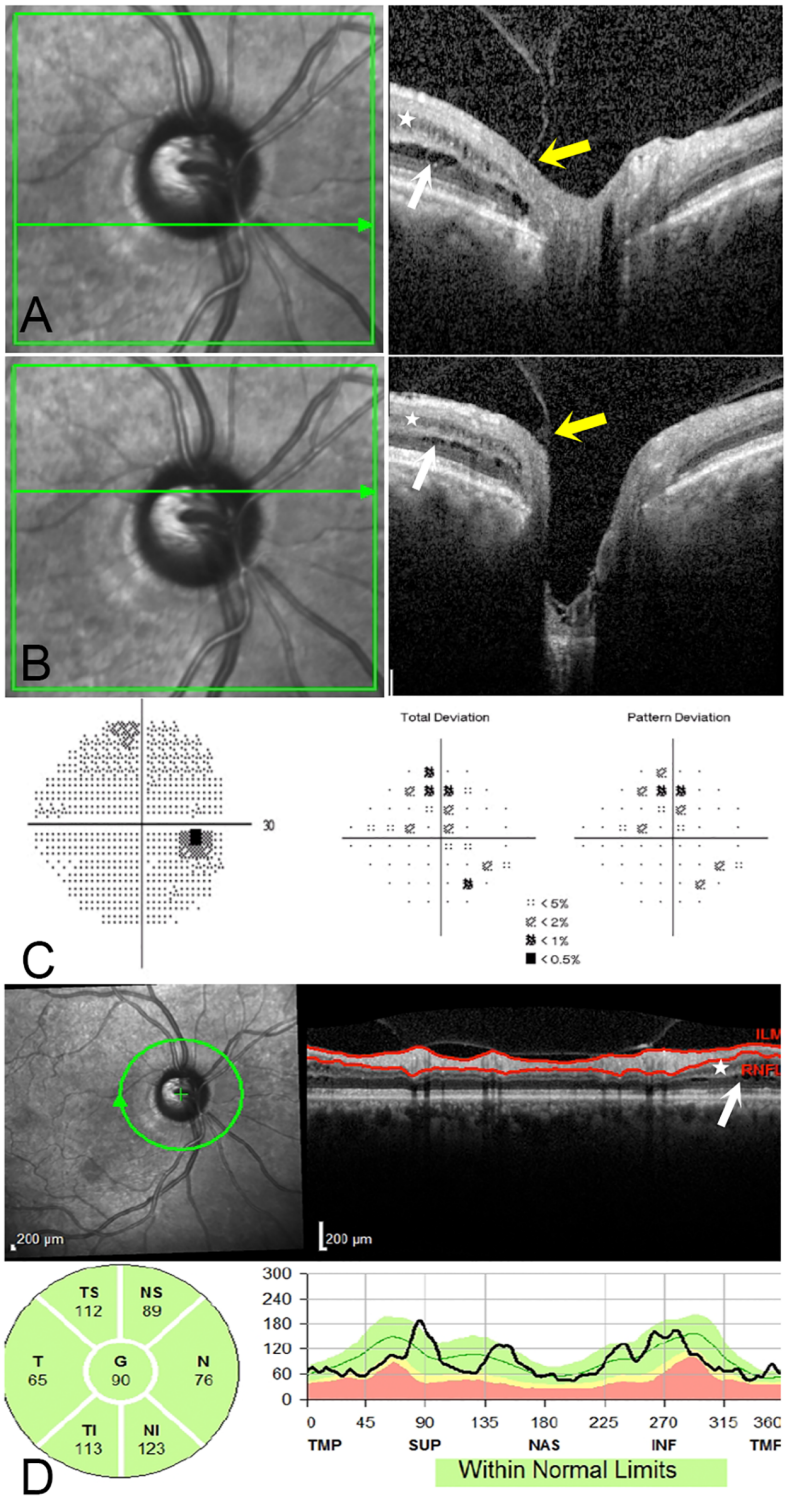
54 year old male with pigmentary OAG with peripapillary retinoschisis and peripapillary atrophy. (3A and 3B) Two horizontal OCT raster scans through the optic nerve head show splitting in the inner nuclear layer (white star) and outer plexiform layer (white arrows). There is a focal area of vitreopapillary traction at the temporal margin of the optic nerve head (yellow arrows). (3C) Humphrey visual field shows mild glaucomatous damage with a MD of -1.99 dB. (3D) Circumpapillary RNFL thickness map and section shows RNFL segmentation sparing the retinoschisis in the inner nuclear layer (white star), outer nuclear and outer plexiform layers (white arrow).

A total of 990 eyes of 495 consecutive glaucoma patients and glaucoma suspects who had undergone ONH raster scan OCT imaging were included. Peripapillary schisis was present in 11 eyes (1.1%) of 7 patients (2 females, 5 males, mean age 64.5 ± 9.2 years, range 52 to 78 years). On initial review, an additional 4 cases had been identified to have uncertain findings but were determined to be normal by expert consensus. Figs 1, 2 and 3 provide representative examples of eyes identified with peripapillary schisis.

### Clinical characteristics

The clinical and demographic characteristics of all subjects in the study, as well as the assoicated imaging findings, are summarized in the Table 1. Of the 7 patients with peripapillary schisis, 3 (42.9%) had primary open angle glaucoma (POAG), 2 (28.6%) were glaucoma suspects, 1 (14.3%) had primary angle closure-glaucoma and 1 (14.3%) had pigmentary glaucoma. The distrubution of clinical diagnosis among eyes with and without peripapillary schisis (979 eyes of 488 patients) is provided in Table 2. Detailed information on all subjects is provided in S1 Table.

**Table 1:** Clinical characteristics of the eleven eyes of seven patients with peripapillary retinal splitting (schisis).

	Age/Gender	Diagnosis	Eye	IOP	Layers Involved	Prior Surgery/Laser	Glaucoma Meds	Other ocular pathology	BCVA	CCT (um)	SEQ (D)	VF MD (dB)	VF PSD	Axial length (mm)	CD Ratio	PPA
1	54M	Pigmentary OAG	OD	15	INL, ONL, OPL	None	Latanoprost	None	20/20	574	-8.75	-1.99	1.88	NA	0.7	Yes
2	78M	POAG	OD	11	NFL, IPL, OPL, ONL	CE	Bimatoprost, Dorzolamide	None	20/20	513	-0.75	0.09	2.02	22.71	0.75	No
3	78M	POAG	OS	11	INL, ONL	CE	Bimatoprost, dorzolamide	None	20/30	511	-1.25	-2.19	2.19	22.76	0.75	No
4	66M	OHT	OD	19	NFL, INL, ONL	None	None	None	20/25	531	-7.75	-0.08	2.98	NA	0.4	Yes
5	66M	OHT	OS	19	NFL, INL, ONL	None	None	None	20/25	543	-8.50	-0.86	1.88	NA	0.3	Yes
6	69F	OHT	OD	17	NFL, ONL	None	Latanoprost	None	20/40	573	-3.50	-1.02	2.26	27.74	0.8	No
7	69F	OHT	OS	14	NFL, ONL	CE	Latanoprost	None	20/25	574	-4.50	-2.83	1.98	27.90	0.8	No
8	67M	CNAG	OS	17	INL, ONL, OPL	LPI, peripheral iridoplasty	Brimonidine, Brinzolamide, Latanoprost	None	20/20	577	+4.75	-1.53	2.49	NA	0.1	No
9	67M	POAG	OD	14	INL, ONL,	Trab, Tube Shunt	Brimonidine	None	20/30	536	-2.75	-21.03	11.47	NA	0.8	Yes
10	67M	POAG	OS	11	NFL, INL, ONL	Trab	None	None	20/30	544	-3.25	-19.65	13.42	NA	0.7	Yes
11	80F	POAG	OS	10	NFL, INL, ONL	Trab	Timolol, Latanoprost	None	20/70	470	-3.25	-27.61	8.98	25.01	0.9	Yes

POAG = primary open angle glaucoma; OHT = ocular hypertension; CNAG = chronic narrow angle glaucoma; CE = cataract extraction; LPI = laser peripheral iridotomy; trab = trabeculectomy; IOP = intraocular pressure; BCVA = best corrected visual acuity; CCT = central corneal thickness; SEQ = spherical equivalent; VF MD = visual field mean deviation; VF PSD = visual field pattern standard deviation; CD Ratio = cup disc ratio; PPA = peripapillary atrophy; NFL = nerve fiber layer; OPL = outer plexiform layer; ONL = outer nuclear layer; INL = inner nuclear layer.

**Table 2:** Distribution of diagnosis in eyes with and without peripapillary retinal splitting (schisis).

Eyes with Peripapillary Schisis	n = 11 eyes
Primary Open Angle Glaucoma	5 (45.4%)
Glaucoma Suspect	4 (36.3%)
Primary Angle Closure Glaucoma	1 (9.1%)
Pigmentary Glaucoma	1 (9.1%)
Eyes without Peripapillary Schisis	n = 979 eyes
Primary Open Angle Glaucoma	481 (49.1%)
Glaucoma Suspect	162 (16.5%)
Chronic Narrow Angle Glaucoma	45 (4.6%)
Pseudoexfoliation Glaucoma	52 (5.3%)
Ocular Hypertension	120 (12.3%)
Uveitic Glaucoma	33 (3.4%)
Pigmentary Open Angle Glaucoma	27 (2.8%)
Other Secondary Open Angle Glaucoma	24 (2.5%)
Pigment Dispersion Syndrome	2 (0.6%)

Mean IOP (mean ± standard deviation) at the time of imaging for these patients was 14.4 ± 3.3 mmHg as compared to 16.3 ± 4.9 for eyes without peripapillary schisis (p = 0.19). For eyes with peripapillary schisis, the mean Humphrey visual field mean deviation (mean ± standard deviation) was –7.16 ± 3.32 dB (median -2.40, interquartile range -0.84 to -7.89) and the mean pattern standard deviation was 4.69 ± 4.37 dB (median 2.16, interquartile range 1.94 to 4.42) which was not significantly different from eyes without peripapillary schisis, at -4.93 ± 7.14 dB (median 2.13, interquartile range -0.25 to -7.17, p = 0.64) and 4.46 ± 3.83 dB (median 2.37, interquartile range 1.73 to 6.43, p = 0.51) respectively.

### OCT and imaging characteristics

Of the 11 eyes, all 11 (100%) had splitting in the ONL, 8 (72.3%) had splitting in the INL, 7 (63.6%) had splitting in RNFL and 3 (27.3%) had splitting in OPL. Two of these 11 eyes (18.1%) also had extension of the schisis cavity into the macula as visualized on OCT. Seven of the 11 (63.6%) eyes had vitreous traction in the peripapillary area visualized on OCT and 6/11 eyes (54.5%) had beta-zone peripapillary atrophy. In contrast, among the 979 eyes without retinoschisis, 13 (1.33%) had vitreous traction in the peripapillary area visualized on OCT (p<0.001) and 245 eyes (25.03%) had beta zone peripapillary atrophy (p = 0.18, Chi-Square test). Eight of the 11 eyes with schisis (72.7%) had glaucomatous optic disc excavation.

### Axial length and refractive status

Among eyes with available axial length measurements (n = 85 eyes), mean axial length (mm) was 25.22 ± 2.54 among eyes with peripapillary schisis and 24.81 ± 2.32 among eyes without peripapilllary schisis (p = 0.195). Spherical equivalent (Dioptres) was -3.6 ± 3.9 in eyes with peripapillary schisis compared to -1.4 ± 3.4 in those without (p = 0.016). Among the 11 eyes with peripapillary retinoschisis, only 2 eyes were highly myopic (spherical equivalent ≤ −8.0 diopters or axial length > 26.0 mm). The other eyes were mildly to moderately myopic and there was one hyperope. Review of the characteristics of peripapillary retinoschisis between the hyperopic and the myopic eyes did not reveal a significant difference.

## Discussion

Using high density OCT scans, centered on the optic nerve, we observed peripapillary retinal splitting in 1.1% of 990 consecutive glaucoma and glaucoma suspect eyes examined in our clinic. We found that eyes with peripapillary schisis were significantly more myopic and more likely to have vitreopapillary traction compared to eyes without peripapillary schisis. However, there was no significant difference in age, IOP, visual field mean deviation, pattern standard deviation, prevalence of beta-zone peripapillary atrophy, vertical cup disk ratio or axial length between the two groups.

Peripapillary and macular schisis has been described in POAG[13], sometimes associated with serous detachment in eyes with a large optic nerve head cup[14], pseudoexfoliation glaucoma [15] and normal tension glaucoma. [8] The differential diagnosis of splitting in our patients includes myopic foveoschisis, tractional retinoschsis and traction on the disc. While our cross-sectional study cannot determine true causality, there have been various theories put forward to explain the pathogenesis of peripapillary schisis in glaucomatous eyes.

Some postulate that the development of micro-holes in the thinned area of the ONH or RNFL may allow liquid vitreous to enter the retina. [5, 7, 16] The presence of the macular schisis in normal-tension glaucoma also supports the micro-holes theory. [8] There has also been suggestion of a communicating bridge connecting the optic disc and the macular retinoschisis region, contraction of the membrane tissue on the optic disc creating a cleft, an optic nerve anomalous excavation functioning similar to a bulb syringe, and entry of vitreous through the lamina cribrosa, which may also be influenced by alterations in pulse or in the intracranial pressure due to changes in posture. [2] [17] [18] [19] [20] None of the eyes in our series had evidence of optic pits on OCT which have been associated with schisis. [2] [21] [22]

The second theory is that increased IOP and optic nerve cupping also may play a role in the development of retinoschisis. [5] [8] [23] It has also been suggested that small changes in axial length accompanying fluctuations in IOP may lead to vitreous traction, with or without microscopic breaks in the inner retina and play a role in schisis formation which has been reported in chronically elevated IOP. [6] [5, 19, 23, 24] None of the eyes with peripapillary schsis had IOP >21, while among eyes without schisis, 104 eyes had IOP >21 at the time of imaging or on their preceding clinic visit. Overall, there was no difference in the IOP among eyes with and without peripapillary schisis. Similar to our results, Hwang et al. [11] did not find any differences in IOP or visual field indices prior to or at the time of peripapillary retinoschisis formation.

Thirdly, microscopic fractures in the ILM could provide a conduit between the posterior cortical vitreous and the subretinal space [25] leading to macular neurosensory detachments. However, in our series, all cases of schisis extended back to the nerve margins suggesting an optic disk origin rather than an underlying ILM abnormality.

Lastly, vitreomacular or vitreopapillary traction secondary to incomplete vitreous separation is another plausible mechanism. [1, 26–30] [31] In our series, 7/11 (63.6%) of the eyes with peripapillary schisis had evidence of adherent vitreous with traction at the nerve in contrast to 13/979 (1.33%) of eyes without peripapillary schisis, while 404/979 (41.3%) eyes without peripapillary schisis had vitreopapillary adhesion without traction. In a series of 13 POAG eyes with microcystic INL changes similar to schisis, Hasegawa et al reported that the vitreous was attached in the area of microcystic INL spaces in 76.9% of eyes. A partial PVD has also been shown to increase RNFL thickness measurements, suggesting the impact of vitreous traction on the inner retinal layers. [32]

With extension into the macula, retinoschisis can impact vision and may warrant surgical intervention. [29] [7] Spontaneous resolution of macular disease as a result of complete vitreous separation has been reported in 10% of eyes with vitreomacular traction [33, 34] and it is possible that a similar course may be seen in glaucomatous eyes with peripapillary schisis leading to an underdetection of schisis.

Ouyang et al described 278 eyes, from 144 normal volunteers (81 females, 63 males) with a mean age of 37.6 years (range 18–74; SD = 15.5 years) that were imaged with the 3DOCT-1000 (Topcon Corp., Tokyo, Japan) using the raster scan protocol with 512 × 128 scans, centered at the optic nerve head to report the prevalence of juxtapapillary pigment epithelium detachments. [35] These were asymptomatic volunteers that had not been diagnosed with any ocular pathology. We performed a post-hoc analysis of this dataset and upon review of images, found that none of the eyes had peripapillary schisis. While this does not cohort is underpowered to serve as a control group and was significantly younger, and predominantly hispanic, the absence of peripapillary schisis is in contrast to the 1.1% prevalence in our glaucoma and glaucoma suspect group.

The prevalence of peripapillary retinoschsis in glaucoma patients and glaucoma suspects has several implications. Firstly, coexisting retinoschisis in a patient with glaucoma may interfere with the reliability of RNFL thickness analysis, as retinoschisis may mask glaucomatous RNFL thinning due to inclusion of the retinoschisis cavities by the RNFL segmentation algorithm causing artifactual thickening and this should be considered when interpreting RNFL thickness measurements. In a recent study of 19 glaucomatous eyes with retinoschisis, the RNFL thickness was 81.6 microns, significantly greater than 69.7 microns in a group of age and visual field mean deviaton matched glaucomatous eyes.[11] It has been reported that peripapillary retinoschisis is most commonly located in the superior quadrant (63.2%), followed by the inferior quadrant (15.8%), nasal quadrant (10.5%) and the superior-nasal area (5.3%). Clinicians should pay attention to the OCT segmentation algorithm to manually verify accurate segmentation and identify any anomalous pathology in the retinal layers. Retinoschisis has been reported in all the inner retinal layers including the RNFL, GCL and IPL. RNFL thickness has been shown to return to baseline levels following resolution of retinoschisis. [11] In our series, all eyes had splitting in the ONL, 72.3% in INL, 63.6% in the RNFL and 27.3% in the OPL.

Secondly, glaucoma patients and glaucoma suspects with vitreous traction should be followed for the development of retinoschisis as this may impact the visual field in the absence of true glaucomatous progression. High density cross sectional raster images through the optic nerve can accurately identify vitreopapillary traction which may be missed on a 3.46 mm diameter circle RNFL scan.

Thirdly, this is also applicable to eyes with peripapillary atrophy as 54.5% of the eyes with schisis in our study had beta-zone peripapillary atrophy. Although we did not use enhanced depth imaging-OCT, it is also possible that the peripapillary atrophy observed was along the spectrum of peripapillary choroidal cavitation which has been associated with schisis. [36] [37, 38] [39] [40, 41]

The majority of patients in our series with peripapillary retinoschisis had POAG (42.9%) followed by glaucoma suspects (28.6%), chronic narrow angle glaucoma (14.3%) and pigmentaty glaucoma (14.3%), suggesting that schisis may occur across the spectrum of the glaucoma continuum and not just in those eyes with advanced disease. Unlike a typical area of retinoschisis found in the peripheral retina, the schisis associated with optic disc pits does not cause an absolute scotoma [19] and thus may not cause changes in the visual field, making detection using imaging more important.

Limitations of our study include its retrospective nature, lack of an adequately powered non-disease group that precludes us from calculating the prevalance and predictive value of peripapillary schisis in glaucoma and glaucoma suspects, and inability to determine true causality in this cross sectional study.

Unique to our investigation, to the best of our knowledge, is the review of a large series of consecutuve glaucoma patients and glaucoma suspects who underwent high resolution cross sectional OCT raster scans to detect peripapillary schisis. Based on these data, we report peripapillary schisis in 1.1% of a large cohort of glaucoma patients and glaucoma-suspects. Evidence of adherent vitreous with traction and peripapillary atrophy was found in a majority of the eyes with schisis. Further long term prospective studies analyzing the clinical course and characteristics of peripapillary retinoschsis are warranted to better elucidate the clinical significance of this pathology.

## Supporting information

S1 Table Supporting data file contains the information of the 990 glaucoma and glaucoma suspect eyes including the 11 eyes with schisis. (XLSX)
